# Current and Maximum Acceptable Travel Times to Primary Care Among US Older Adults

**DOI:** 10.1001/jamanetworkopen.2025.45280

**Published:** 2025-11-24

**Authors:** Tabasa Ozawa, Ying Liu, Soeren Mattke

**Affiliations:** 1 USC Brain Health Observatory, University of Southern California, Los Angeles

## Abstract

This survey study examines thresholds of current and maximum acceptable travel times to primary care clinicians among older adults in the US.

## Introduction

Older adults in the US have high levels of primary care access; 91.0% have a usual primary care clinician (PCC),^1^ and more than three-quarters travel 30 minutes or less to a PCC’s office.^2^ Yet there are concerns that the declining PCC-population ratio^3^ and hospital closures and mergers^4^ could lead to longer travel times. We investigated the threshold of travel time at which it would become a barrier to primary care access and for which subgroups.

## Methods

The Biomedical Research Alliance of New York Institutional Review Board approved this survey study. Informed consent was obtained electronically. We followed the AAPOR reporting guideline.

Between February 1 and 28, 2025, members of the Understanding America Study (UAS) (a survey panel of >14 000 participants aged ≥18 years) were invited to participate in a survey about mode of transportation, current travel time to primary care, and maximum travel time before delaying or forgoing care (eMethods in Supplement 1). Prior surveys from the same panel collected self-rated health, self-reported urbanicity, and other demographic data. Analysis was restricted to participants aged 50 years or older.

We used logistic regression to examine factors associated with willingness to increase current travel time. Factors, selected based on reported transportation barriers to health care access,^5^ included age, sex, marital status, race and ethnicity, education, household income, urbanicity, mode of transportation, current travel time, and self-rated health. Two-sided *P* < .05 was significant; analyses were conducted using Stata, version 16 (StataCorp LLC). As a robustness check for missing covariates, we repeated the analysis with multiple imputation.

## Results

Of 6119 individuals invited, 4545 (74.3%) completed the survey. We excluded 130 (2.9%) with no PCC and 369 (8.1%) with missing covariates, resulting in 4046 respondents (mean [SD] age, 65.3 [9.3] years; 2288 females [56.5%] and 1758 males [43.5%]). Most respondents reported having a PCC (4383 [96.4%]) and traveling to primary care by car (3818 [94.4%]) (Table). Most (3327 [82.2%]) reported traveling 30 minutes or less, and only 103 (2.6%) traveled more than 60 minutes (Figure). Mean (SD) travel time was 20 (16) minutes overall and 19 (15), 18 (15), and 27 (20) minutes for urban, suburban, and rural residents, respectively.

**Table.** Study Sample Characteristics and Association With Any Increase Between Current and Maximum Travel Times

Characteristic	No. (%) of respondents (N = 4046)	AOR (95% CI)
Age, y		
50-64	1957 (48.4)	1 [Reference]
65-74	1344 (33.2)	1.39 (1.16-1.67)
≥75	745 (18.4)	1.39 (1.10-1.74)
Sex		
Female	2288 (56.5)	0.96 (0.81-1.13)
Male	1758 (43.5)	1 [Reference]
Marital status		
Married and cohabitating	2327 (57.5)	1 [Reference]
Other	1719 (42.5)	0.92 (0.77-1.10)
Yearly household income, $		
≤39 999	1186 (29.3)	1 [Reference]
40 000-99 999	1604 (39.6)	1.67 (1.37-2.04)
≥100 000	1256 (31.0)	2.30 (1.78-2.97)
Race and ethnicity^a^		
Black or African American	443 (10.9)	0.46 (0.37-0.59)
Hispanic, Latino, or Spanish	230 (5.7)	0.53 (0.39-0.72)
White	3021 (74.7)	1 [Reference]
Other	352 (8.7)	0.47 (0.36-0.61)
Educational attainment		
High school or less	773 (19.1)	1 [Reference]
Some college	1463 (36.2)	1.20 (0.97-1.47)
Bachelor degree or more	1810 (44.7)	1.63 (1.30-2.05)
Residence		
Urban	1592 (39.3)	1 [Reference]
Suburban	1545 (38.2)	1.32 (1.10-1.60)
Rural and other	909 (22.5)	1.54 (1.23-1.93)
Mode of transportation to primary care		
Car	3818 (94.4)	1 [Reference]
Other	228 (5.6)	1.16 (0.84-1.61)
Self-reported health		
Excellent, very good, or good	3246 (80.2)	1 [Reference]
Fair or poor	800 (19.8)	1.22 (1.00-1.49)
Current travel time^b^	NA	0.55 (0.51-0.60)

Abbreviations: AOR, adjusted odds ratio; NA, not applicable.

^a^ In the Understanding America Study, race is categorized as American Indian or Alaska Native, Asian, Black or African American, Native Hawaiian or Other Pacific Islander, or White. Ethnicity (Hispanic, Latino, or Spanish) is asked separately. To ensure adequate cell sizes, we have recoded American Indian or Alaska Native, Asian, and Native Hawaiian or Other Pacific Islander as other race or ethnicity. Race and ethnicity were included in the regression analysis based on previous research on transportation barriers to health care access.

^b^ Time intervals were treated as a continuous variable for the logistic regression.

**Figure.**
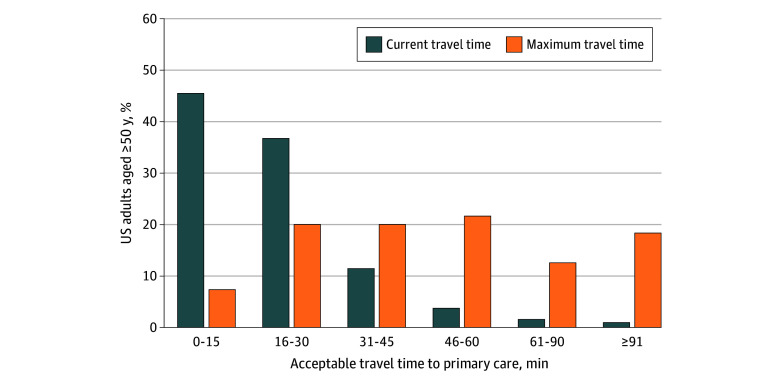
Current and Maximum Acceptable Travel Times to Primary Care Among Adults Aged 50 or Older in the US

Mean (SD) maximum acceptable travel time was 53 (31) minutes overall and 48 (31), 53 (30), and 61 (30) minutes for urban, suburban, and rural residents, respectively. Most respondents (3116 [77.0%]) said they would increase travel time before delaying or forgoing care, with a mean (SD) increase of 33 (30) minutes.

Logistic regression revealed that older age, higher income, White race, higher education, nonurban residence, and shorter current travel time were associated with willingness to increase travel time (Table). Substantial differences in willingness to travel were seen between higher-income (≥$100 000; 1073 of 1256 [predicted probability, 83.0%]) and lower-income (≤$39 000; 767 of 1186 [predicted probability, 69.9%]) individuals (adjusted odds ratio [AOR], 2.30 [95% CI, 1.78-2.97]) and between Black (261 of 443 [predicted probability, 69.9%]; AOR, 0.46 [0.37-0.59]) and Hispanic (149 of 230 [predicted probability, 67.5%]; AOR, 0.53 [0.39-0.72]) individuals compared with White individuals (2470 of 3021 [predicted probability, 80.4%]). The results were similar with multiple imputation.

## Discussion

Most older adults (96.4%) reported currently having a PCC, and 82.2% traveled 30 minutes or less to see them; 77.0% preferred taking longer trips over delaying or forgoing care. This study’s main limitation is that it relied on stated preference, which may differ from actual behavior. Self-reported data may also include recall bias. Insurance status was unavailable, and correction for multiple testing was not applied. Our results may not generalize to younger age groups, other types of medical services, and individuals excluded from the UAS, including speakers of languages other than English or Spanish and institutionalized individuals.

Our findings suggest that substantial increases in travel time could discourage primary care use among individuals with lower income and education, racial and ethnic minority individuals, urban residents, and individuals with longer travel time. Interventions aimed at alleviating transportation barriers to health care access should consider these populations.

## Notes

Supplement 1.
eMethods

Supplement 2.
Data Sharing Statement
