# Supplementary material for: Current and Maximum Acceptable Travel Times to Primary Care Among US Older Adults
Source: JAMA Netw Open. 2025 Nov 24;8(11):e2545280. doi: 10.1001/jamanetworkopen.2025.45280 (PMC12645317; doi:10.1001/jamanetworkopen.2025.45280)
Supplement: Supplement 1. — eMethods [file jamanetwopen-e2545280-s001.pdf]

Supplemental Online Content

Ozawa T, Liu Y, Mattke S. Current and maximum acceptable travel times to primary care among US older adults. *JAMA Netw Open*. 2025;8(11):e2545280.
doi:10.1001/jamanetworkopen.2025.45280

eMethods

This supplemental material has been provided by the authors to give readers additional information about their work.

© 2025 Ozawa T et al. *JAMA Network Open*.

eMethods

A. Information on the Understanding America Study

The Understanding America Study (UAS) is a nationally representative, probability-based Internet panel maintained by the University of Southern California's Center for Economic and Social Research. There are approximately 15,000 panelists aged 18 and older, who participate in both longitudinal and cross-sectional online surveys. To maximize representativeness, all surveys are available in English and Spanish, and respondents are provided with an internet-connected tablet if they do not have their own device and Internet access.

Respondents are invited to join the UAS using Address-Based Sampling (ABS), in which addresses are randomly selected from postal records representing all addresses in the country. All individuals in the sampled household aged 18 and older are eligible to join the study. The UAS also includes address-based samples of California and Los Angeles County, as well as special-purpose samples of Native Americans and Los Angeles County families with young children. Non-probability samples are given a zero weight in UAS surveys, and analysis can be restricted to the nationwide sample to exclude oversampled populations.

Demographic information is collected in the My Household questionnaire, which is fielded to all new respondents and can be reviewed and updated quarterly by all respondents. Respondents typically receive one or two surveys per month, including the core survey fielded every two years, and various other surveys and experiments. Panelists receive $20 for every 30 minutes of survey time, paid monthly via funds added to a prepaid card. The surveys have high response rates, typically averaging 70-80 percent.

More information on the UAS and survey data can be found on the UAS website (https://uasdata.usc.edu/index.php). Researchers can access the data at no cost by signing the Data User Agreement. Restricted access data, including linkages to external data from the Social Security Administration and Centers for Medicare and Medicaid Services are also available through LINKAGE, administered by the National Institute on Aging.

B. Survey questions about travel time and mode to primary care

Q1 How long do you typically travel to see your primary care doctor?

1 0-15 minutes
2 16-30 minutes
3 31-45 minutes
4 46-60 minutes
5 61-90 minutes
6 More than 90 minutes
7 I don't have a primary care doctor

[If Q1≠7]

© 2025 Ozawa T et al. *JAMA Network Open*.

Q2 What is the maximum time that you would travel to see a primary care doctor, before you would delay or forgo medical care?

1 0-15 minutes
2 16-30 minutes
3 31-45 minutes
4 46-60 minutes
5 61-90 minutes
6 More than 90 minutes

[If Q1≠7]

Q3 How do you typically travel to see your primary care doctor?

1 By car
2 By public transportation
3 Other means

C. Survey questions for other covariates

Q1 Would you say your health is excellent, very good, good, fair, or poor?

1 Excellent
2 Very good
3 Good
4 Fair
5 Poor

Q2 Which of the following comes closest to describing the region where you now live?

(If you live in more than one location, please choose the description that best describes

your main residence.)

1 Large city
2 Small city
3 Suburb near a city
4 Small town or village
5 Rural - Agricultural / Ranch/ Farm area
6 Rural but not Agricultural / Ranch / Farm
7 Reservation
8 Other, please specify:

© 2025 Ozawa T et al. *JAMA Network Open*.
