# Supplementary material for: Current and Maximum Acceptable Travel Times to Primary Care Among US Older Adults
Source: JAMA Netw Open. 2025 Nov 24;8(11):e2545280. doi: 10.1001/jamanetworkopen.2025.45280 (PMC12645317; doi:10.1001/jamanetworkopen.2025.45280)
Supplement: Supplement 2. — Data Sharing Statement [file jamanetwopen-e2545280-s002.pdf]

Data Sharing Statement

Ozawa. Current and Maximum Acceptable Travel Times to Primary Care Among US Older Adults. *JAMA Netw Open*. Published November 24, 2025.
doi:10.1001/jamanetworkopen.2025.45280

Data
Data available: Yes
Data types: Deidentified participant data
How to access data: The raw survey data will be placed on the UAS website once the paper is published
When available: With publication

Supporting Documents
Document types: None

Additional Information
Who can access the data: Available under DUA
Types of analyses: Any purpose
Mechanisms of data availability: without investigator support
